# State Control of Midwives

**Published:** 1898-09

**Authors:** 


### STATE CONTROL OF MIDWIVES.

YELLOW journalism is responsible for a great deal of harm. At the same time it does occasionally accomplish some good. In the midst of war's turmoils one of the exponents of frothy sensationalism in New York city took time to call attention to a great evil which not only exists, but flourishes in this state.

The newspaper referred to secured an interview with Martin Thorn, the murderer of Guldensuppe, a few days before he paid the penalty of his crime in the electric chair, and secured from him some facts regarding the professional career of Mrs. Nack, Thorne's accomplice, which are cruel enough to send a thrill of horror through every right-minded human being. It will suffice for the time being to give a brief résumé of Martin Thorn's statements. He said that Mrs. Nack was a professional abortionist; that her house was full of women all the time under treatment; that the babies were "disposed" of.

"How?" asked the interviewer. "How were they disposed of?"

"Oh! in various ways," answered Thorn.

He then said that when a case was complicated and of unusual seriousness a "regular doctor" was called in, and that if the real inside facts of Mrs. Nack's business were published, together with the names of some of her clients and medical assistants there would be a great sensation in New York city.

These statements were made by a man who stood on the threshold of certain death. They were made seriously, calmly and without any evidence of being inspired by feelings of revenge.

Attention was called during the interview to a trap door in Mrs. Nack's house, leading into a sewer. The kitchen stove was also spoken of in a way that left no doubt in the minds of those who heard the statements that the babies' bodies were burned, and the fragments and ashes thrown through the trap door into the sewer, whence they were swept into the East river and so on to the sea.

These facts in this case, being almost the dying words of a man who was one of the actors in a most barbarous crime, directs attention forcibly to a condition of affairs which, we must admit, obtains in almost every large city in the union—the trafficking in human life by midwives. There are many legitimate midwives who perform their duties conscientiously; but there are others.

There should be a remedy for this. There is a remedy. It may be remedied by placing midwives under state control, the same as physicians.

Why should midwives be exempt from state examination and state license? True, they are now under certificate of examining boards in some counties, but they should be placed under state license which would afford uniformity in standard. Then, and then only will it be possible to weed out the discreditable ones and make more secure a profession which has to deal with two human lives—the mother's and the child's.

